# Changes of cyprinid fishery resources in Lake Biwa over 57 years: association with multiple stressors and restoration measures

**DOI:** 10.1007/s00442-025-05762-9

**Published:** 2025-07-02

**Authors:** Shin-ichiro S. Matsuzaki, Keiichi Fukaya, Kohji Mabuchi, Takeshi Kikko, Noriko Takamura

**Affiliations:** 1 Biodiversity Division, National Institute for Environmental Studies, 16-2 Onogawa, Tsukuba, Ibaraki 305-8506 Japan; 2 Lake Biwa Branch Office, National Institute for Environmental Studies, 5-34 Yanagasaki, Otsu, Shiga 520-0022 Japan; 3 Faculty of Agriculture, Kindai University, Nakamachi 3327-204, Nara, Nara 631-8505 Japan; 4 Lake Suwa Environmental Research Center, Osachi-gongen-chou 4-11-51, Okaya, Nagano 394-0081 Japan

**Keywords:** Anthropogenic driver, Cyprinidae, Habitat modification, Invasion, Life-history strategy, Spawning habitat

## Abstract

Multiple stressors can threaten the resources of inland fisheries, but responses to these stressors may differ among fish species. We used a long-term fishery dataset to quantify the CPUE trajectories of eight cyprinid taxa in the south basin of Lake Biwa that have been stressed by lakeshore development, artificial water-level regulation, exotic fish species, and climate change. A Bayesian state-space model revealed that the CPUEs of all eight taxa substantially declined from 1966 to 2022. For *Opsariichthys uncirostris*, *Ischikauia steenackeri*, *Sarcocheilichthys* spp., *Cyprinus carpio*, and *Carassius* spp., the average population growth rates decreased from 1976 to 1991, when lakeshore development occurred and largemouth bass populations increased dramatically. For the three remaining taxa (*Zacco platypus*, *Gnathopogon caerulescens*, and *Squalidus* spp.), the average population growth rates decreased from 1992 to 2022, when new water-level regulations were implemented and bluegill populations increased substantially. The former five taxa exhibited longer maximum body length and life span, later maturation, and higher fecundity than the latter three taxa. This suggests that life-history traits may determine how species respond to different stressors. Unlike the effects of these three abrupt stressors, those of climate, a gradual stressor, were negative for seven taxa, although not statistically significant. We also found that the population growth rates of *Gnathopogon caerulescens* and *Ischikauia steenackeri* increased recently after implementation of restoration measures (fishing moratorium and stocking of paddy-reared/captive-bred individuals). Our results suggest that cumulative anthropogenic stressors depleted the resource of cyprinids but that the outcome of current restoration measures may be positive.

**Supplementary Information:**

The online version contains supplementary material available at 10.1007/s00442-025-05762-9.

## Introduction

Inland capture fisheries play important roles by ensuring food security, providing livelihoods, and sustaining cultural identities in both developed and developing countries (Allan et al. 2005; Cooke et al. 2016; Welcomme et al. 2010). Many inland fisheries involve multiple species. They are generally characterized by small-scale/household-based activities, and the bulk of the catch is consumed locally (Beard et al. 2011; Welcomme et al. 2010). Despite the ecological and socioeconomic importance of inland fisheries, inland waters have been severely impacted by multiple stressors associated with human activities (Dudgeon et al. 2006). However, because inland fisheries are not a high priority for government agencies, they have received little attention from government officials, and hence there are neither reliable historical data that could serve as a baseline nor long-term monitoring data. The absence of these data could lead to the “invisible collapse” of fishery resources, community, and culture (Noble et al. 2016).

Lakes support valuable inland fisheries for numerous species. However, conflicts with multiple human demands for purposes such as irrigation, hydropower generation, flood protection, reclamation, and recreational use, have led to a serious deterioration of lake ecosystems (Dudgeon et al. 2006; Kao et al. 2020). Lakeshore development and artificial water-level regulation, which are two major stressors associated with these demands, can seriously impact the habitats fish use for feeding, spawning, and refuge (Bryan and Scarnecchia 1992; Chhor et al. 2020; Jeppesen et al. 2015; Strayer and Findlay 2010). Furthermore, lakes are among the ecosystems most susceptible to invasions by exotic species, because once established, they can easily spread through connected natural and artificial waterways (Ricciardi and MacIsaac 2011). Commercial and sports fish (mostly piscivores), which have been intentionally and unintentionally released for recreational purposes, can affect native fish species directly and indirectly (Matsuzaki and Kadoya 2015). These multiple stressors may deplete fishery resources by acting individually, simultaneously, or cumulatively. Unlike marine systems, where the primary threat is internal (i.e., overfishing), threats to inland fisheries are known to be largely external (Beard et al. 2011; Cooke et al. 2016). Unlike abrupt stressors that cause substantial impacts over a relatively short period of time, climate change is a gradual stressor linked to long-term environmental changes, and it can adversely affect fish and fisheries through a variety of mechanisms, including shifts in suitable habitats and changes of primary production (O’Reilly et al. 2003; Pörtner and Peck 2010). However, it is unclear whether individual fish species respond similarly or differently to multiple stressors, including climate change (Gutowsky et al. 2019; Lange et al. 2018). This lack of clarity highlights the need to disentangle the effects of multiple stressors at the levels of both species and communities.

**Fig. 1:**
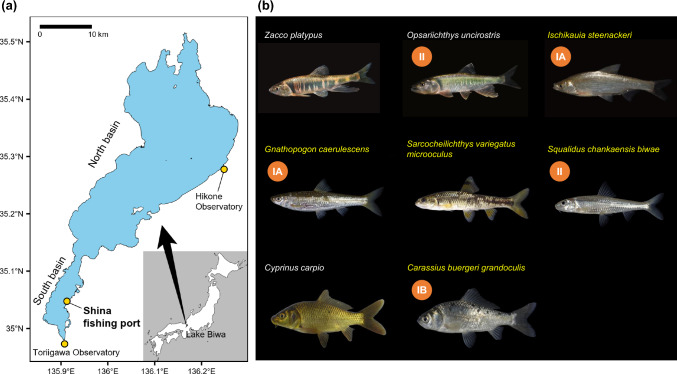
**a** Maps of the south basin of Lake Biwa and the fishing port of the Shina Fisheries Cooperative Association (SFCA). The Toriigawa Observatory for monitoring of the water level in Lake Biwa is located at the outlet of the lake. The Seta River Weir, which is the gate to control water level, is located ~ 4 km downstream from the Toriigawa Observatory. The Hikone Observatory is a local meteorological station operated by the Japan Meteorological Agency. **b** Photographs of the eight studied cyprinid taxa. Endemic species are shown in yellow font. Red circles show the conservation status as determined by the Ministry of the Environment (IA: critically endangered, IB: endangered, II: vulnerable). Photo credit: Yuichi Kano, Yusuke Miyazaki, and Mizuki Matsunuma

Lake Biwa (Fig. 1) harbors 46 native species and subspecies of freshwater fish, of which 17 species and subspecies are endemic (Kawanabe et al. 2012; Yuma et al. 1998). The cyprinid fishery is an important fishery in the lake (Maehata 2012). Local people eat cyprinid fishes caught in Lake Biwa and have developed a distinctive food culture. One of the most famous traditional foods for special occasions is fermented fish dishes, called ‘*nare-zushi*’, made from endemic cyprinid fishes, such as *Carassius buergeri grandoculis*, *Carassius cuvieri*, *Ischikauia steenackeri*, and *Gnathopogon caerulescens* (Maehata 2012). Many of these cyprinid fishes utilize the south basin of Lake Biwa—a small (approximately 8% of the total area), shallow, productive habitat that serves as a crucial spawning and nursery grounds—often referred to as the “cradle of Lake Biwa” (Miura 1966; Renaissance of Lake Biwa and Yodo River Basin 2005). However, the south basin has been greatly affected by human activities, and the catches of cyprinid fish have been declining for the last three or four decades. Among cyprinid fishery species, the Japanese Species Red List of the Ministry of Environment of Japan has already listed *Ischikauia steenackeri* and *Gnathopogon caerulescens* as critically endangered, *Carassius buergeri grandoculis* and *Carassius cuvieri* as endangered, and *Opsariichthys uncirostris* as vulnerable.

**Fig. 2:**
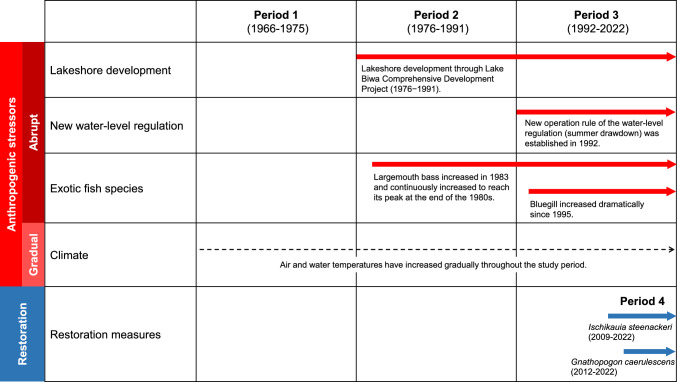
Definition of the three time periods when different anthropogenic stressors acted in the south basin of Lake Biwa. Period 4 is added (2006–2022 and 2012–2022, respectively) to evaluate the effect of restoration measures for *Ischikauia steenackeri* and *Gnathopogon caerulescens*

Four major stressors are considered to have fundamentally disrupted the Lake Biwa community composition, ecosystem functions, and local livelihoods (Fig. 2) (Fujioka 2013; Kira and Uda 1990; Nishino et al. 2011; Yamamoto et al. 2006). The first, lakeshore development, involved the constructions of embankments for water use, flood control, and roads along the lakeshore during 1972–1997 (Tatsumi 2012). Lakeshore development caused a rapid, large loss of the emergent macrophyte beds, particularly along the eastern shore of the south basin (Ohtsuka et al. 1996). The second was a new water level operation rule in 1992 (hereafter “new water-level regulation”), which requires a low water-level during June–October to prevent flooding and make use of lake water. This summer water-level drawdown can affect cyprinid species that spawn in early summer and decrease the connectivity with waterbodies around the lake (Maehata and Moriyasu 2012). The third was the introduction of two exotic species: the piscivorous largemouth bass (*Micropterus salmoides*) and omnivorous bluegill (*Lepomis macrochirus*), which increased dramatically after the late 1980s and the mid 1990s, respectively (Nakai 2020). These two exotic species threaten cyprinid fishes through predation and competition. The final stressor is climate change, because the temperature of the surface water of Lake Biwa has risen gradually. Narita et al. (2024) have reported a negative relationship between offshore bottom temperatures and the catch of the benthic goby (*Gymnogobius isaza*). Although these multiple pressures can significantly threaten cyprinid fish, Fujioka (2013) has speculated that lakeshore development and exotic species mainly caused the decrease of *Carassius auratus grandoculis* and *Carassius cuvieri*, whereas water-level regulation caused the decrease of *Gnathopogon caerulescens*. In contrast, Yamamoto et al. (2006) have reported that new water-level regulation affected *Carassius* spp. and *Cyprinus carpio* populations. One major reason for the disparate conclusions about stressor effects is that they are based on only catch data, which depend on fishing effort and are spatially aggregated across individual sectors where fishing efforts differ. Few studies have examined how anthropogenic stressors can affect other minor or lower-valued cyprinid species and the whole cyprinid community in Lake Biwa. A community-wide approach based on different long-term data is necessary for the Lake Biwa fishery to recover.

In addition, numerous restoration efforts have been undertaken to minimize the decline of fishery resources in Lake Biwa. These efforts have included artificial hatching and release, prohibiting fishing during the spawning season, rearing juveniles in rice paddy fields, planting reed seedling along the lakeshore, removing exotic species of fish and overgrown macrophytes. These management interventions have varied in scale, intensity, and budget over time (Fujioka 2013; Kawanabe et al. 2012). Quantifying the long-term trajectories of cyprinid fishery resources is crucial, not only to assess the effectiveness of past and current management strategies, but also to better motivate restoration managers and practitioners.

The Shina fisheries cooperative association (SFCA) has recorded both catch and effort data that provide a unique opportunity to trace the long-term trajectories of cyprinid fishery resources at individual fishery level. Our goal was to quantify the long-term population trends of eight cyprinid fishery taxa using this unique dataset collected from 1966 to 2022 (Fig. 1) and to examine how their population growth rates changed in response to the four major stressors mentioned above. We applied a Bayesian state-space model to estimate the long-term catch-per-unit-effort (CPUE) of each taxon. To investigate the effects of multiple stressors on individual cyprinid taxa, we identified three distinct time periods when there were differences in the levels of abrupt stressors—lakeshore development, new water-level regulation, and exotic fish species—and then we determined whether the population growth rates of individual taxa varied among those periods. For the gradual stressor, climate, we assessed the effect of the temperature anomaly on population growth rates during each period to distinguish the effect of temperature from the impacts of the abrupt stressors. We also conducted exploratory post hoc analyses to determine whether differences of life-history traits among taxa depended on the period of their population declines. The answer may help disentangle differences between the impacts of multiple stressors. Furthermore, we focused on recent, multiple measures to restore *Gnathopogon caerulescens* and *Ischikauia steenackeri*, and we assessed whether their population growth rates had responded positively to these interventions (Fig. 2).

## Materials and methods

### Long-term fishery data collection

Lake Biwa (surface area 670 km^2^) is located entirely within Shiga Prefecture in Japan, and the mean depths of its north and south basins are very different (44 m and 3.5 m, respectively) (Fig. 1a). The SFCA, which is located at Kusatsu City by the south basin, has caught mainly cyprinid fishes using gill nets and *Eri* (Fig. S1), a unique and traditional large fixed-trap net. We obtained long-term catch data (1966–2022) from the SFCA. We used annual catch data of eight cyprinid fishery taxa (Fig. 1b). The data for *Carassius* spp. included catches of *Carassius buergeri grandoculis*, *Carassius auratus* complex, and *Carassius cuvieri*; the data for *Sarcocheilichthys* spp. included catches of *Sarcocheilichthys variegatus microoculus*, *Sarcocheilichthys variegatus variegatus*, and *Sarcocheilichthys biwaensis*; and the data for *Squalidus* spp. included catches of *Squalidus chankaensis biwae* and *Squalidus japonicus japonicus*.

We obtained four potential sources of effort data from the SFCA: the number of fishers (regular members), the number of *Eri*, the number of fisher households using *Eri*, and the number of fisher households using gillnets. We finally decided to use the number of fishers and confirmed its validity (Appendix S1, Fig. S2).

### Defining time periods in relation to multiple stressors and restoration measures rates

To explore the impacts of the anthropogenic stressors (lakeshore development, new water-level regulation, exotic fish species, and climate) and restoration measures on cyprinid taxa, we estimated their population growth rates in relation to time periods classified based on differences in stressors and restoration measures (LeTourneux et al. 2022). To define the time periods, we investigated when the relevant events occurred in the south basin of Lake Biwa.

### Anthropogenic stressors

We categorized lakeshore development, new water-level regulation, and exotic fish species as abrupt stressors, and we classified climate as a gradual stressor.

#### Lakeshore development

The Lake Biwa Comprehensive Development Project (1972–1997) was implemented in and around the lake to stabilize the supply of lake waters to the downstream areas and to reduce the adverse effects of droughts and floods (Kira and Uda 1990; Nishino et al. 2011). During 1976–1991, continuous embankments with a road were constructed along 57 km of the lake’s shoreline, ports and harbors were renewed, and the irrigation system was improved for irrigation of paddy fields around the lake (Fujioka 2013). Because of these extensive lakeshore developments, many of natural shorelines with emergent vegetation disappeared and became artificial shorelines, such as concrete beach and stone masonry (Ohtsuka et al. 1996; Tatsumi 2012).

#### New water-level regulation

Water-level control has been conducted by weir operation in Lake Biwa since the 1900s, and the summer water level was maintained at a Biwako Basic Surface Level (B.S.L.) of 0 cm after construction and installation of the Seta River Weir in 1961. During the Lake Biwa Comprehensive Development Project, new rules for regulating the water level (Regulation for Seta River Weir Operation) were established in 1992 to prevent flood damage and make better use of the lake’s water (Maehata and Moriyasu 2012; Nishino et al. 2011). Under the rules, the B.S.L. is to be + 30 cm from October 15 to June 14, − 20 cm from June 15 to August 31 (rainy season), and − 30 cm from September 1 to October 14 (typhoon season). We collected long-term monitoring data of the actual water level (1968–2019) recorded at 06:00 at the Toriigawa Observatory (Fig. 1a) ~ 4 km north of the Seta River Weir (Nakanishi et al. 2022). These data clearly showed that the water level during May–July, which are spawning seasons of many cyprinid fishes, have been lower after the implementation of new water-level regulations (Fig. S3).

#### Exotic fish species

Two exotic fish, largemouth bass and bluegill, are considered to substantially affect the cyprinids of Lake Biwa. Largemouth bass and bluegill were first caught in 1974 and 1965, respectively. The largemouth population increased suddenly in 1983 and continued to increase to reach a peak at the end of the 1980s (Nakai 2020; Nishino et al. 2011). Since 1990, the abundance of largemouth bass has apparently declined. Since 1995, the bluegill population has dramatically increased in the littoral zone. The SFCA has caught largemouth bass and bluegill since 1983, but only the combined total catch of these two species has been recorded. We calculated the CPUE of total exotic fish species as a metric of their relative abundance. The CPUE first increased in the latter half of the1980s and increased dramatically in the 2000s (Fig. S4).

Based on this stressor information, we defined three time periods (Fig. 2). Period 1 (1966–1975) was the “baseline” before the three stressors acted. Period 2 (1976–1991) was the time of lakeshore development through the Lake Biwa Comprehensive Development Project. During this period, the largemouth bass population also increased dramatically. Period 3 (1992–2022) began after the start of the new water-level regulations. During this period, the bluegill population also increased substantially.

#### Climate

We initially attempted to collect surface-water temperature data from the Water Information System (Ministry of Land, Infrastructure, Transport and Tourism 2020), which includes temperatures measured monthly at a pelagic site in the south basin (Yanagasaki-oki, Fig. S5a). Although those data were available on a monthly basis from 1966 to 2022, the sampling frequency between 1966 and 1979 was too sparse—only four times per year—to calculate reliable annual means. As an alternative, we obtained long-term annual air temperatures monitored at the Hikone Meteorological Observatory in the north basin (Fig. 1a) from the Japan Meteorological Agency’s web-accessible database (Fig. S5A). Given the strong correlation between annual mean water temperatures and air temperatures from 1980 to 2022 (*r*^2^ = 0.81, Fig. S5B), we used air temperature in the subsequent analyses as a reliable indicator of the effects of global warming from 1966 to 2022.

The temperature data showed a long-term upward trend. To prevent confounding the effects of the three periods mentioned above with the effects of rising temperature, we focused in our analysis on the deviation from the average temperature (i.e., the temperature anomaly) for each period.

### Restoration measures for *Gnathopogon caerulescens* and *Ischikauia steenackeri*

We assessed the effectiveness of recent restoration measures for two cyprinid species. Shiga Prefecture has conducted two restoration programs simultaneously for *Gnathopogon caerulescens* since 2012 (Kikko 2020; Kikko et al. 2017). One program has involved rearing of hatched larvae in paddy fields adjacent to the south basin of Lake Biwa and release of juveniles to the lake. The other program has been a moratorium on commercial fishing of *Gnathopogon caerulescens* during the spawning season and was first implemented during 2012 in Ibanaiko Lagoon, which is connected to the north basin of Lake Biwa. The moratorium has been expanded to the entire lake since 2016. For *Ischikauia steenackeri*, the stock enhancement center of Lake Biwa has released captive-breed individuals into the south basin since 2009.

An additional period associated with the restoration measures was therefore defined as a period 4 (2012–2022 and 2009–2022) for *Gnathopogon caerulescens* and *Ischikauia steenackeri*, respectively, to assess whether the restoration measures were successful. For *Gnathopogon caerulescens* and *Ischikauia steenackeri*, period 3 was redefined as 1992–2011 and 1992–2008, respectively, to ensure that there are no years were duplicated in the defined periods.

### Estimating long-term CPUE trajectories and average growth rates per period

We developed a Bayesian state-space model to estimate the trajectory of CPUE (a measure of relative abundance) from 1966 to 2022, the average population growth rates per period, and the effect of temperature anomalies on the annual population growth rate of the cyprinid fish taxa. The model was formulated hierarchically to enable analysis of the data for the eight fish taxa simultaneously so that we could estimate the parameters for each taxon and the average parameters at the community level.

#### Model specification

Let ${y}_{it}$ be the annual catch (expressed in grams) of taxon *i* in year *t*, with 1966 corresponding to *t* = 1. With ${x}_{it}$ as the CPUE of taxon *i* in year *t* and ${z}_{t}$ as the effort (the number of fishers) in year *t*, we modeled the time series of annual catch for *t* > 1 and CPUE for *t* > 1 as follows:

$$\text{log}{y}_{it}\sim N(\text{log}{x}_{it}+\text{log}{z}_{t},{\sigma }_{\left(y\right)i}^{2})$$

$$\text{log}{x}_{it}\sim N\left(\text{log}{x}_{i,t-1}+{R}_{it}+{S}_{it},{\sigma }_{\left(x\right)i}^{2}\right),$$

where ${\sigma }_{\left(y\right)i}^{2}$ and ${\sigma }_{\left(x\right)i}^{2}$ are the variances of the log annual catch of taxon *i* and the process variance of the log CPUE of taxon *i*, respectively. ${R}_{it}$ represents the taxon- and period-specific average log population growth rates, and ${S}_{it}$ represents the fluctuation of the log population growth rate around its taxon- and period-specific average explained by temperature anomaly. For taxon *i*, excluding *Gnathopogon caerulescens* and *Ischikauia steenackeri*, the average log population growth rate was expressed as:

$${R}_{it}={\beta }_{1,i}+{\beta }_{2,i}{P}_{2, t-1}+{\beta }_{3,i}{P}_{3,t-1},$$

where ${P}_{2,t}$ and ${P}_{3,t}$ are variables that indicate that year *t* belongs to period 2 and period 3, respectively. The average log population growth rate for period 1 of taxon *i* was thus expressed as ${\beta }_{1,i}$, and the average log population growth rates for period 2 and period 3 were expressed as ${\beta }_{1,i}+{\beta }_{2,i}$ and ${\beta }_{1,i}+{\beta }_{3,i}$, respectively.

Period 4 was defined for *Gnathopogon caerulescens* and *Ischikauia steenackeri*, and the average log population growth rate was expressed as:

$$ R_{it} = \beta_{1,i} + \beta_{2,i} P_{2,t - 1} + \beta_{3,i} P^{\prime}_{3,i,t - 1} + \beta_{4,i} P_{4,i,t - 1} . $$

where ${P}_{4,i,t}$ is an indicator variable indicating that year *t* belongs to period 4 for taxon *i*. Note that for *Gnathopogon caerulescens* and *Ischikauia steenackeri*, the indicator variable for period 3 is changed from ${P}_{3,t}$ to $P^{\prime}_{3,i,t}$, which represents period 3 only for *Gnathopogon caerulescens* and *Ischikauia steenackeri*.

${S}_{it}$ was expressed as:

$${S}_{it}={\gamma }_{i}{T}_{i, t-1},$$

where ${T}_{i, t-1}$ represents the temperature anomaly and ${\gamma }_{i}$ represents its effect on taxon *i*. As mentioned above, the temperature anomaly was defined as the deviation from the average temperature during a specific period. Since the periods differed between taxa, the temperature anomaly includes a subscript *i* for the taxon.

Because some of the effort data were missing, we modeled the time series of the effort ${z}_{t}$ for *t* ≥ 1 and its expected values for *t* > 1as follows:

$${z}_{t}\sim \text{Poisson}\left({\lambda }_{t}\right)$$

$$\text{log}{\lambda }_{t}\sim N(\text{log}{\lambda }_{t-1},{\sigma }_{(\lambda )}^{2}),$$

where ${\lambda }_{t}$ is the expected value of the effort in year *t*, and ${\sigma }_{(\lambda )}^{2}$ is the process variance of the expected log effort.

For the Bayesian inference component of the model, we specified prior distributions for unknown parameters and variables. To estimate taxon-specific average population growth rates and their community-level averages, we specified a multivariate normal prior distribution with an unknown mean vector and covariance matrix for ${\beta }_{1,i}$, ${\beta }_{2,i}$, ${\beta }_{3,i}$, and ${\gamma }_{i}$. We specified vague prior distributions for other parameters and the initial values, $\text{log}{x}_{i,1}$ and $\text{log}{\lambda }_{1}$.

#### Inference

Because the catch time series model is specified on a logarithmic scale and thus cannot be fitted to zero-valued data, we replaced data with an annual catch of zero with the minimum value of the catch record (50).

We fitted the model with JAGS version 4.3.2 software (Plummer 2003) via the jagsUI version 1.6.2 package (Kellner 2024) in R version 4.4.2 software (R Core Team 2024). We ran six independent Markov chains, each with 1000 burn-in iterations followed by 100,000 iterations thinned at intervals of 100 to obtain the posterior samples. We assessed the convergence of the posterior with the $\widehat{R}$ statistic. The convergence was achieved at the recommended level ($\widehat{R}<1.1$) for all parameters and unknown variables.

We estimated the average log population growth rates per period for each fish taxon as derived quantities based on posterior estimates of ${\beta }_{1,i}$, ${\beta }_{2,i}$, ${\beta }_{3,i}$, and ${\beta }_{4,i}$. We estimated community-level averages of the log population growth rates per period as posterior estimates of the mean vector specified for ${\beta }_{1,i}$, ${\beta }_{2,i}$, and ${\beta }_{3,i}$. The community-level average of the temperature effect ${\gamma }_{i}$ was estimated in the same way.

The JAGS code used to run the model can be found in Appendix S2.

### Life-history trait analysis

We divided the eight cyprinid taxa into two groups based on whether the posterior median of the average log population growth rates decreased considerably from period 1 to period 2 or from period 2 to period 3. We examined whether the groups shared similar life-history traits to elucidate how cyprinid taxa responded to different stressors. From Matsuzaki et al. (2016), we extracted data for four life-history traits: (1) maximum total body length (cm); (2) female age at maturation (years); (3) longevity, the maximum potential life span (category 1: 2−4 years, category 2: 5−10 years, category 3: > 10 years); (4) fecundity, the total number of eggs per breeding season (category 1: 1000−10,000, category 2: 10,000−100,000, category 3: > 100,000). We used generalized linear mixed models (GLMMs) with Gaussian error distributions to determine whether there were differences of trait values between the two groups. To account for trait similarities among taxa due to shared ancestry, we included subfamily (Cyprininae, Oxygastrinae, and Gobioninae; classified by Saitoh (2014)) as a random factor to perform a phylogenetic correction. The GLMMs were fitted using the *lmer* function in the *lme4* package (Bates et al. 2015) using maximum likelihood estimation. Ordinal traits were converted to ranks using the *rank* function. We used R software 4.2.2.

## Results

### Long-term CPUE trajectories of eight cyprinid taxa

**Fig. 3:**
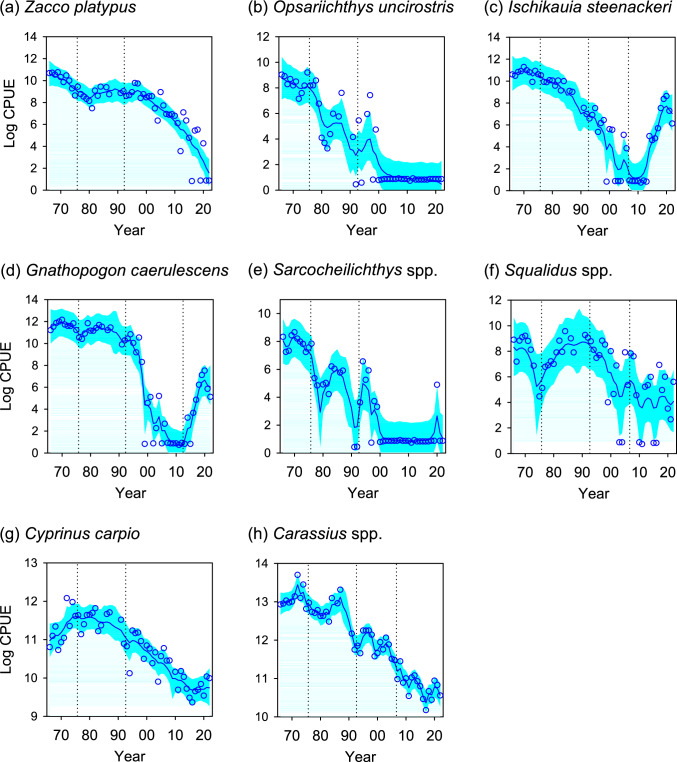
Estimated long-term trends in the log-transformed CPUEs of eight cyprinid taxa over 57 years (period 1: 1966–1975, period 2: 1976–1991, period 3: 1992–2022) in the south basin of Lake Biwa (**a**–**h**). For *Ischikauia steenackeri* (**c**) and *Gnathopogon caerulescens* (**d**), period 4 was added (2006–2022 and 2012–2022, respectively) to evaluate the effect of restoration measures. Solid blue lines indicate posterior median values, and cyan shaded areas indicate 95% posterior credible intervals. Blue circles indicate raw log-transformed CPUEs

Estimated long-term trajectories of the CPUE of the eight cyprinid taxa in the south basin of Lake Biwa indicated that all populations of all eight taxa declined over 57 years. The onset of decline varied among taxa (Fig. 3). The CPUEs of *Opsariichthys uncirostris*, *Ischikauia steenackeri*, *Sarcocheilichthys* spp., *Cyprinus carpio*, and *Carassius* spp. started declining from period 2 (Fig. 3b, c, e, g, and h) and continued to decline during period 3. The CPUEs of *Zacco platypus*, *Gnathopogon caerulescens*, and *Squalidus* spp*.* started declining from period 3 (Fig. 3a, d, and f). The CPUEs of *Ischikauia steenackeri* and *Gnathopogon caerulescens* increased slightly during period 4 (Fig. 3c and d).

### Responses of population growth rates to multiple stressors

**Fig. 4:**
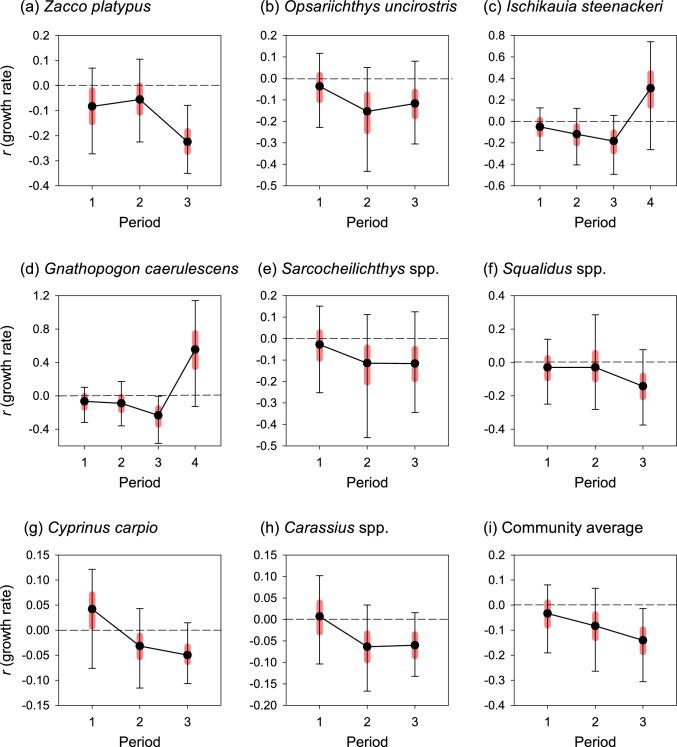
The average log population growth rates (**a**–**h**) and community average growth rate (**i**) during the three periods (period 1: 1966–1975, period 2: 1976–1991, period 3: 1992–2022). Period 4 was added for *Ischikauia steenackeri* (**c**) and *Gnathopogon caerulescens* (**d**) (2006–2022 and 2012–2022, respectively) to evaluate the effect of restoration measures. Solid circles are posterior median values, black error bars indicate 95% posterior credible intervals, and light red bars indicate 50% posterior credible intervals

Although the estimates of the average log population growth rates per period were associated with considerable uncertainty, they characterized the differences in the CPUE trajectories among cyprinid taxa well. In *Opsariichthys uncirostris*, *Ischikauia steenackeri*, *Sarcocheilichthys* spp., *Cyprinus carpio*, and *Carassius* spp., the posterior median of the average log population growth rate decreased considerably and became negative during period 2 (Fig. 4b, c, e, g, and h). The posterior probabilities of decline exceeded 0.84 for all five taxa (Table S1), and the negative growth rates persisted during period 3; thus, the populations of these taxa have continually declined since period 2. In contrast, the population growth rates of *Zacco platypus*, *Gnathopogon caerulescens*, and *Squalidus* spp*.* decreased to negative values during period 3 (Fig. 4a, d, and f), and the posterior probabilities of decline exceeded 0.92 for all three taxa (Table S1); therefore, the declines of the populations of these taxa were most apparent during period 3 (Fig. 4a, d, and f). For *Ischikauia steenackeri* and *Gnathopogon caerulescens*, the average log population growth rate increased and became positive during period 4 (Fig. 4c and d), indicating that the population increased during period 4. The posterior probabilities of population growth of *Ischikauia steenackeri* and *Gnathopogon caerulescens* were 0.88 and 0.95, respectively (Table S1). The community average of the population growth rates of the eight cyprinid taxa consistently decreased during the three periods (Fig. 4i).

**Fig. 5:**
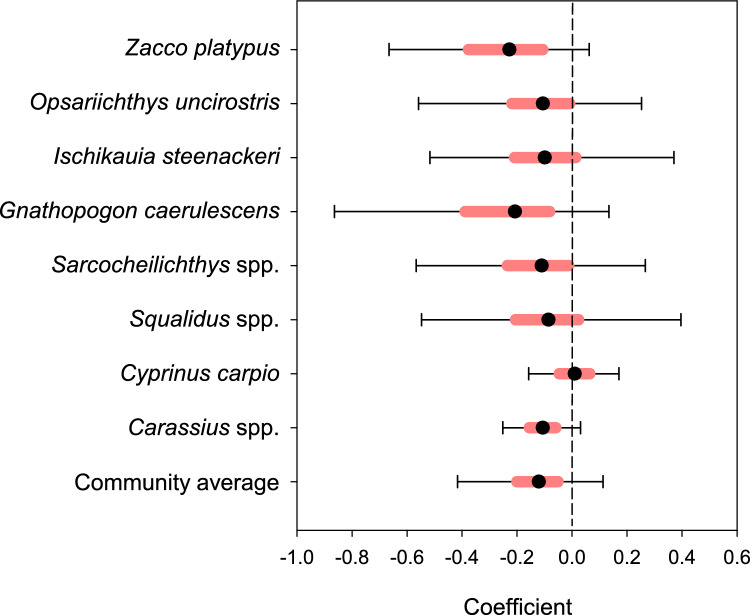
The coefficients of the effects of climate (air temperature) on eight cyprinid taxa and community average over 57 years (1966–2022). Solid circles are posterior median values, black error bars indicate 95% posterior credible intervals, and light red bars indicate 50% posterior credible intervals

The effects of temperature on log population growth rates were negative for all the cyprinid taxa, except for *Cyprinus carpio* as well as for the community average, but the effects were not statistically significant (i.e., the 95% posterior credible intervals overlapped zero, Fig. 5).

### Life-history trait analyses

**Fig. 6:**
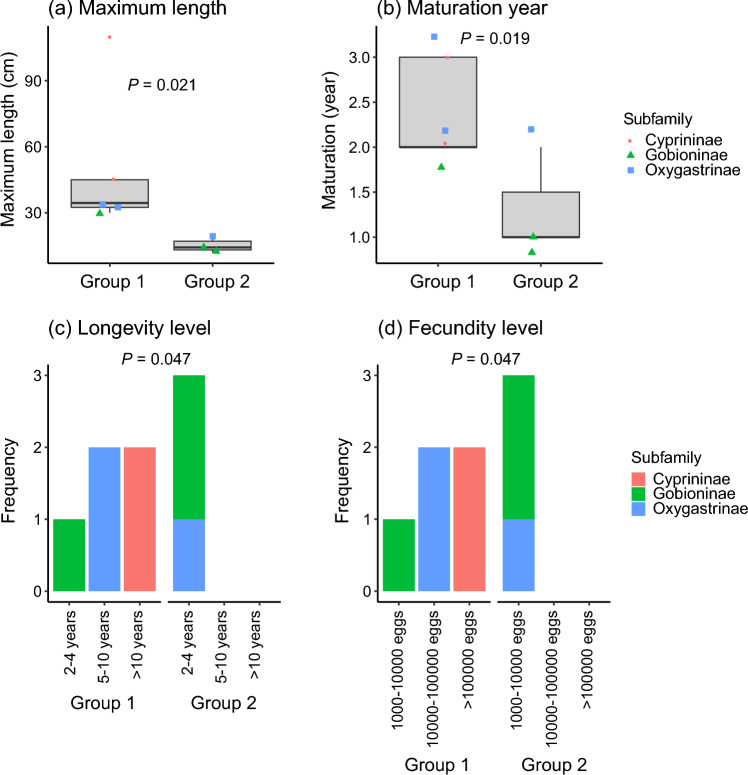
Differences of four life-history traits between the group of five cyprinid taxa for which the CPUE declined during period 2 (group 1, *n* = 5) and the group of three taxa for which the CPUE declined during period 3 (group 2, *n* = 3). Continuous traits (**a**, **b**) and ordinal traits (**c**, **d**) are shown as boxplots and frequency-distribution plots, respectively. Different color symbols or bars represent different subfamilies. Boxplots show the median and the 25th and 75th percentiles. The *P*-values were calculated using a GLMM

A group of five cyprinid taxa experienced a remarkable decrease of the posterior median of the average log population growth rates from period 1 to period 2 (Group 1, *N* = 5). The GLMMs revealed that Group 1 had a significantly longer maximum body length (Fig. 6a) and later maturation (Fig. 6b) than the group of the other three cyprinid taxa (Group 2, *N* = 3), which experienced a remarkable decrease of those rates from period 2 to period 3. Although the category values of longevity and fecundity were identical among the eight taxa, the GLMMs revealed that Group 1 had a significantly longer life span (Fig. 6c) and higher fecundity (Fig. 6d) compared to Group 2. Phylogenetic signals appeared to be minimal across all traits because representatives of all three subfamilies occurred in both groups.

## Discussion

Overall, our analysis of long-term fishery data demonstrated that multiple stressors decreased all eight cyprinid fishery resources in the south basin from 1966 to 2022. However, the timing of the decreases divided the eight taxa into two groups: one group showed remarkable declines of their average log population growth rates during period 2; the other group showed remarkable declines during period 3. We also found the differences in life-history traits between the two groups. Climate adversely affected seven of the eight cyprinid taxa, although the effects were not statistically significant. Our results suggested that each cyprinid taxa might be affected differently by specific stressors, but the cumulative effect of multiple stressors may be a decrease of the resource of the whole cyprinid community. Our findings importantly highlighted the potential for recovery of the populations of two of the taxa through restoration efforts.

### Potential impacts of lakeshore development and largemouth bass (period 2)

The CPUEs of five cyprinid taxa (*Opsariichthys uncirostris*, *Ischikauia steenackeri*, *Sarcocheilichthys* spp*.*, *Cyprinus carpio*, and *Carassius* spp.) substantially declined during period 2 and continued to decline during period 3. This suggests that lakeshore development and/or largemouth bass may have been the primary stressors that decreased their resources. According to the Lake Biwa Comprehensive Development Project (1976–1991), 73% of the total length of the lakeshore of the south basin was changed to artificial lakeshores, including concrete beaches and stone masonry (Kira and Uda 1990; Tatsumi 2012). The area along the shoreline of emergent vegetation, dominated by *Phragmites australis*, therefore decreased from 72.8 ha (1955–1962) to 24.6 ha (1995) (Ohtsuka et al. 1996). Cyprinid species make extensively use of lakeshores diurnally, seasonally, and ontogenetically. *Cyprinus carpio* and *Carassius* spp. spawn in reed beds; *Sarcocheilichthys* spp. spawns on live unionid mussels in the lakeshore; *Opsariichthys uncirostris* and *Ischikauia steenackeri* use shores as a feeding habitat (Yuma et al. 1998). The severe loss of shore vegetation shores and physically complex habitats may thus have decreased the abundance of cyprinid species (Bryan and Scarnecchia 1992; Chhor et al. 2020; Itakura et al. 2015; Strayer and Findlay 2010).

Largemouth bass may have affected cyprinid taxa during period 2. Because there are few native piscivores in Japan (Watanabe 2012), direct predation by largemouth bass can have a strong negative impact on the population growth rates of cyprinid species (Maezono and Miyashita 2003; Matsuzaki and Kadoya 2015). Because *Opsariichthys uncirostris* is the only piscivorous cyprinid species in Japan, competition for food between this species and largemouth bass may have occurred. Tsunoda et al. (2016) have reported that the growth rates of younger classes of *Opsariichthys uncirostris* as well as the standard length of adults decreased between the 1960s and 2013, but Imamura et al. (2024) observed no significant changes in the mean size of *Opsariichthys uncirostris* during the time interval 1995–2014, which corresponds to period 3. Food availability associated with lakeshore development and the invasion of largemouth bass might therefore have contributed to the decline of their population growth rates during period 2. In addition, lakeshore development might have boosted the largemouth bass population during period 2. Largemouth bass often prefers developed shorelines, which can increase shade and shelter (Taillon and Fox 2004), and shores with steeper slopes, which enables it to forage more effectively in open water and access deep-water refugia (Middaugh et al. 2013).

The five cyprinid taxa that declined during period 2 had life histories characterized by large body size, long life, late maturation, and high fecundity, but with the caveat that we could not clearly differentiate between fecundity and longevity because these traits were classified into identical categories. Such taxa are classified as periodic-type species, which are characterized by low juvenile survivorship and are associated with highly seasonal environments (Winemiller 2005). Periodic species use multiple habitats during different life cycle stages, and in particular, they reproduce under stable conditions Periodic species are therefore prone to endangerment by habitat fragmentation and degradation (Olden et al. 2006; Winemiller 2005). In addition, small differences of mortality during early life stages caused by a variety of factors, including food abundance and predator density, can potentially have a huge influence on their population dynamics and growth (Hitt et al. 2020; Winemiller 2005). Our life-history trait analysis therefore suggests that both lakeshore development and largemouth bass may modify the environmental conditions for spawning and decrease juvenile survival.

Our results are seemingly inconsistent with a previous finding that the new water-level regulation decreased the number of eggs and larvae of *Carassius* spp. and *Cyprinus carpio* in the reed zone (Yamamoto et al. 2006). One of the main reasons for this discrepancy is that Yamamoto et al. (2006) compared those variables between only1964 and 1996 (i.e., they did not include any year during period 2). Our results suggest that the populations of *Carassius* spp. and *Cyprinus carpio* were affected mainly by lakeshore development and/or largemouth bass in period 2 before the water-level regulation (period 3).

### Potential impacts of new water-level regulations and bluegill (period 3)

The CPUEs of the three cyprinid taxa (*Zacco platypus*, *Gnathopogon caerulescens*, and *Squalidus* spp*.*) declined dramatically during period 3. This result suggests that the new water-level regulation and/or bluegill may have negatively impacted these three taxa. Since 1992, summer water levels have been intentionally lowered to maintain at B.S.L of − 20 cm, which is 30–40 cm lower than in previous years (Maehata and Moriyasu 2012). Even such a small change can cause a reduction and dewatering of cyprinid spawning areas. In particular, *Gnathopogon caerulescens* is called as the willow minnow because, in early summer, they enter very shallow water and lay eggs around the roots of willow trees and the submerged parts of reeds (Kikko et al. 2013; Mabuchi et al. 2023). Even if they succeed in spawning, subsequent water-level drawdown will dry up their eggs (Gaboury and Patalas 1984).

The three fish taxa with relatively small body sizes, short generation timea, early maturation, and low batch fecundity were classified as opportunistic strategy types (Winemiller 2005). Opportunistic-type species are expected to be favored in environments characterized by frequent and intense disturbances (e.g., harsh and unstable hydrology). Stabilizing seasonal variations and homogenizing flow variations by dam regulation can decrease the abundance of opportunistic-type species and select for life history strategies adapted to more stable and predictable environments (McManamay and Frimpong 2015). Our trait analysis suggests that stabilizing the water level and consecutive water-level drawdowns during early summer may have contributed to the observed declines of the populations of this functional group.

Bluegill can affect cyprinid taxa both directly and indirectly. In Japan, bluegill feeds on a variety of prey items, and its diet changes ontogenetically (Azuma 1992). Taniguchi (2012) has reported that bluegill competes with native species for food and substantially decreases the feeding and growth rates of native species. Direct predation on eggs is another pathway, since large bluegill consume fish eggs (Azuma 1992; Katano et al. 2003). Several cyprinid such as *Squalidus chankaensis biwae* scatter their sticky eggs on muddy sand substrates in the littoral areas without defending the eggs after spawning (Katano et al. 2003). These eggs can be easily consumed by bluegill (Yokogawa 2018).

Along with the new water-level drawdown regulation, an extreme drought in 1994 facilitated recovery of submerged macrophytes in the south basin of Lake Biwa (Nishino et al. 2011). The area covered by macrophytes subsequently expanded and reached 6, 29, and 43 km^2^ by 1994, 2000, and 2002, respectively (Haga et al. 2007). Because Sakai et al. (2020) have reported a positive correlation between the abundances of young bluegill and submerged macrophytes in the south basin of Lake Biwa during 2010 and 2018, macrophyte overgrowth might have increased bluegill populations. Bluegill prefers dense submerged macrophyte beds as a feeding habitat and to reduce the risk of predation by largemouth bass (Dewey et al. 1997). In contrast, macrophyte overgrowth has caused oxygen depletion at the bottom (Ishikawa et al. 2019). *Zacco platypus* is reported to be more vulnerable to hypoxic waters than other cyprinids (Yamamoto 1991). The new water-level regulation and associated macrophyte overgrowth combined with bluegill abundance may therefore have affected cyprinid taxa in a complex way during period 3.

The results of a previous study that used carbon and nitrogen stable isotopes (Okuda et al. 2012) have suggested that the predatory piscivore *Opsariichthys uncirostris* consumes *Gnathopogon caerulescens*, *Zacco platypus*, and *Squalidus* spp. The substantial decline of *Opsariichthys uncirostris* during period 2 would therefore lead to the expectation that its prey taxa would increase during periods 2 or 3. However, all three prey taxa instead experienced significant declines during period 3. The implication is that predator–prey interactions may not be particularly strong among these taxa. However, we cannot rule out the possibility that competitive interactions among the cyprinid taxa may have shifted over time.

### Positive outcome of restoration measures (period 4)

The increase of the average population growth rate of *Gnathopogon caerulescens* during period 4 is consistent with recent reports of substantial expansions of spawning areas across the lakeshore of the south basin (Mabuchi et al. 2023) and the increase of the percentage of individuals over 1 year of age since 2018 (Shiga Prefectural Fisheries Experimental Station 2023). Although their fishing moratorium that has been imposed during the spawning season may be effective in reducing mortality from commercial and recreational fishing on the most valuable adults in terms of reproductive output (van Overzee and Rijnsdorp 2015), the moratorium alone may be an insufficient safeguard in the context of the multiple ongoing anthropogenic pressures on the fishery (Chen et al. 2020). A combination of stocking of larger sized larvae and juveniles reared in paddy fields may help *Gnathopogon caerulescens* populations recover. Kikko et al. (2013) have reported that the growth and survival rates of *Gnathopogon caerulescens* larvae and juveniles reared in paddy fields are higher than those reared in lakes and lagoons because of the warmer water temperatures and higher food abundances in the former. In addition to these restoration measures, Shiga Prefecture has removed largemouth bass and bluegill from the south basin since 2011 using an electrofishing boat, which is particularly effective in capturing large largemouth bass (Yoshioka et al. 2012). Considering that the CPUEs of other cyprinid taxa have not recovered since 2011, measures to specifically restore *Gnathopogon caerulescens* may have been more effective.

Although the population growth rate of *Ischikauia steenackeri* also increased during period 4, the recapture rate of marked individuals has been reported to exceed 70% (The Stock Enhancement Center of Lake Biwa 2023). This implies that the survival rate of captive-bred individuals is high, but the population is not increasing by natural spawning. A restoration measure to increase the spawning area and the survival rate of larvae and juveniles needs to be identified.

### Potential impacts of climate change

Climate affected negatively, but not significantly, the growth rates of seven cyprinid taxa (Fig. 5). These results may indicate that there is a tendency for the impact of gradual warming to exacerbate the adverse effects of abrupt stressors such as lakeshore development, new water-level regulations, and the introduction of exotic fish species, on cyprinid taxa. Our results do not provide strong evidence of an adverse effect of climate on the population growth of cyprinid taxa. However, our results are consistent with those of previous studies of the effects of warming on Lake Biwa and other lakes. Although warm-water fish such as cyprinids are physiologically less vulnerable to warming compared to cold-water species, raising temperatures can lead to higher rates of food consumption to gain enough energy to meet increasing metabolic costs (Hartman and Jensen 2017; Pörtner and Peck 2010). Primary production in Lake Biwa has decreased since the 1990s, and this decline has been related to warming in addition to a reduction of nutrient inputs (Kazama et al. 2024). In Lake Tanganyika, a 20% reduction of primary production due to warming caused a 30% decrease of fish yields (O’Reilly et al. 2003). Climate-induced changes of food availability and food-web interactions can thus increase competition for food among cyprinid species and other fish species (Hartman and Jensen 2017). The weakness of the relationships that we observed may have reflected our use of annual mean air temperatures. Climate impacts can vary spatially, vertically, and seasonally, and extreme events like summer heatwaves and severe droughts can significantly influence ecosystems. Further research is therefore urgently needed to investigate the direct and indirect effects of climate variability on cyprinid fish resources.

### Limitations

There were at least four limitations to this study. First, we used 1966–1975 as the reference period. However, many lagoons, which are spawning and nursery habitats for cyprinid species, were reclaimed as paddy fields to meet the increased demand for food after World War II, and the total lagoonal area decreased from 2902 ha in 1940 to 703 ha in 1960 (Nishino et al. 2011). The CPUEs of cyprinid taxa during period 1 may not represent a valid baseline because they may have declined to some extent due to loss of lagoonal habitat. Second, we did not assess the effect of overfishing. While the fishing efforts of the SFCA have declined over time, the CPUEs of all eight cyprinid taxa have not recovered since the 1980s. We believe that the effect of overfishing on cyprinid fishery resources has probably been small compared to the impacts of the three stressors we analyzed, but testing this hypothesis will require detailed analysis of other fishery data or fishery-independent monitoring data. Third, we acknowledge that our approach did not quantify the relative impacts of individual stressors. Incorporating more detailed, quantitative data on stressors could inform management strategies by helping to clarify the extent to which factors such as lakeshore development or water-level drawdown affect cyprinid fishery resources. Alternative approaches, such as stable isotope analyses and size/age measurements of historical museum specimens (Vander Zanden et al. 2003), should be considered in future studies to elucidate the specific long-term impacts of exotic fishes—especially largemouth bass and bluegill—on each of the eight cyprinid taxa. Fourth, we did not examine the potential influences of changing precipitation patterns and de-eutrophication. These factors may be important for understanding how fishery resource management can be effectively integrated with water level and water quality management under future climate change scenarios.

### Management implications

Our results highlight the need for management based on species-specific responses to multiple stressors to sustain the cyprinid fishery in Lake Biwa. Mitigating the impacts of lakeshore development and water-level regulation may be desirable but seriously conflicts with water use and flood control as the climate changes. There are several potential options to overcome these tradeoffs. One is restoring hydrological connectivity between the lake and other nearby habitats, including rivers, lagoons, paddy fields, and ditches. Maintaining and connecting diverse habitat mosaics, particularly between rice paddies and Lake Biwa, is needed to increase spawning and feeding habitats and provide refugia from predatory exotic fish (Fujioka 2013; Kikko et al. 2024). Another is restoring and constructing reed zones as spawning grounds and nursery habitats. Their proper spatial arrangement could greatly enhance the connectivity among habitats (Matsuzaki et al. 2011b). Artificial submerged structures can also provide additional fish habitat and refugia and increase the availability of prey (Chhor et al. 2020), but these options should also consider the increase of largemouth bass and bluegill.

Suppressing both largemouth bass and bluegill will be necessary, because substantial evidence has suggested that whole-lake-scale removal of these species can effectively restore native fish communities (Weidel et al. 2007). Shiga Prefecture has intensified efforts to remove them by financially supporting their capture since 1999, by prohibiting the releases of exotic fish species in recreational fishing since 2002, and by proactive removal with an electrofishing boat since 2011 (Nakai 2020; Yoshioka et al. 2012). According to Shiga Prefecture annual reports, the estimated biomasses of largemouth bass and bluegill decreased twofold and sevenfold from the 2000s to the 2020s, respectively. However, the abundances of these species can quickly rebound. A new exotic piscivore, channel catfish (*Ictalurus punctatus*), was first captured in the south basin in 2007 and a total of 44 individuals were captured by 2020 (Ishizaki et al. 2022). Because channel catfish has already impacted fishery resources in some Japanese lakes (Matsuzaki et al. 2011a), its early eradication should be a priority.

Many measures to restore cyprinid fishery resources have currently been undertaken in Lake Biwa. A monitoring scheme should be carefully designed to correctly and effectively evaluate the effect of past and current interventions. The adverse effect of increasing temperature on *Gnathopogon caerulescens* should be taken seriously (Fig. 5), because future warming could undermine efforts to facilitate recovery of its population via restoration efforts. This possibility underscores the need for adaptive restoration strategies to address future climate change, such as adjusting the timing and location of stocking paddy-reared or captive-bred individuals.

## Supplementary Information

Below is the link to the electronic supplementary material.

Supplementary file1 (DOCX 1197 KB)

## Data Availability

Data are available from the authors upon reasonable request.
