# Supplementary material for: Changes of cyprinid fishery resources in Lake Biwa over 57 years: association with multiple stressors and restoration measures
Source: Oecologia. 2025 Jul 2;207(7):121. doi: 10.1007/s00442-025-05762-9 (PMC12222436; doi:10.1007/s00442-025-05762-9)
Supplement: Supplementary file 1 — Supplementary file1 (DOCX 1197 KB) [file 442_2025_5762_MOESM1_ESM.docx]

**Supporting information**

**Changes of cyprinid fishery resources in Lake Biwa over 57 years: association with multiple stressors and restoration measures**

Shin-ichiro S. Matsuzaki^a,*^, Keiichi Fukaya^a^, Kohji Mabuchi^b^, Takeshi Kikko^c^, Noriko Takamura^a,d^

^a^*Biodiversity Division, National Institute for Environmental Studies, 16-2 Onogawa, Tsukuba, Ibaraki, 305-8506 Japan*

^b^*Lake Biwa Branch Office, National Institute for Environmental Studies, 5-34 Yanagasaki, Otsu, Shiga, 520-0022 Japan*

^c^*Faculty of Agriculture, Kindai University, Nakamachi 3327-204, Nara, Nara, 631-8505 Japan*

^d^*Lake Suwa Environmental Research Center, Osachi-gongen-chou 4-11-51, Okaya, Nagano, 394-0081 Japan*

***Correspondence**: Shin-ichiro S. Matsuzaki (E-mail: matsuzakiss@nies.go.jp)

**Appendix S1 Selection of effort data**

The number of fishers was highly and positively correlated with the other three effort data (Appendix Fig. S1a−c). To confirm the effectiveness of the number of fishers, we also analyzed the relationship between the number of fisher households and the total annual fishing days in Kusatsu City (including fisheries cooperative associations in Shina, Yamada, and Yabase) using the Shiga Prefectural Annual Statistics of Fishery and Aquaculture Production. The correlation was highly positive, although the period was limited ton 1970−2001 (Appendix Fig. S1d). We thus chose the number of fishers (1966−2022) as an indicator of fishing effort

**Appendix S2 The JAGS code used in this study**

model {

### Observation model

for (t in 1:N_year) {

for (i in 1:N_species) {

# Log catch

log_y[i, t] ~ dnorm(mu_log_y[i, t], tau_y[i])

mu_log_y[i, t] <- log_x[i, t] + log(z[t])

}

# Effort

z[t] ~ dpois(lambda[t])

log(lambda[t]) <- log_lambda[t]

}

### Process model

for (t in 2:N_year) {

# i = 1: Gnathopogon caerulescens

log_x[1, t] ~ dnorm(log_x[1, t - 1] + R[1, t] + S[1, t], tau_x[1])

R[1, t] <- beta1[1] +

beta2[1] * phase2[t - 1] +

beta3[1] * phase3[t - 1] +

beta4[1] * phase4_Gc[t - 1]

S[1, t] <- gamma[1] * temperature[1, t - 1]

# i = 2: Ischikauia steenackeri

log_x[2, t] ~ dnorm(log_x[2, t - 1] + R[2, t] + S[2, t], tau_x[2])

R[2, t] <- beta1[2] +

beta2[2] * phase2[t - 1] +

beta3[2] * phase3_Is[t - 1] +

beta4[2] * phase4_Is[t - 1]

S[2, t] <- gamma[2] * temperature[2, t - 1]

# Others

for (i in 3:N_species) {

log_x[i, t] ~ dnorm(log_x[i, t - 1] + R[i, t] + S[i, t], tau_x[i])

R[i, t] <- beta1[i] +

beta2[i] * phase2[t - 1] +

beta3[i] * phase3[t - 1] +

beta3[i] * phase4_Gc[t - 1]

S[i, t] <- gamma[i] * temperature[i, t - 1]

}

log_lambda[t] ~ dnorm(log_lambda[t - 1], tau_lambda)

}

### Priors

for (i in 1:N_species) {

log_x[i, 1] ~ dnorm(0, prior_prec)

tau_y[i] <- pow(sigma_y[i], -2)

tau_x[i] <- pow(sigma_x[i], -2)

sigma_y[i] ~ dunif(0, prior_ulim)

sigma_x[i] ~ dunif(0, prior_ulim)

spec_eff[i, (1:N_par)] ~ dmnorm.vcov(Mu, Sigma)

beta1[i] <- spec_eff[i, 1]

beta2[i] <- spec_eff[i, 2]

beta3[i] <- spec_eff[i, 3]

gamma[i] <- spec_eff[i, 4]

}

for(i in 1:N_par){

Mu[i] ~ dnorm(0, prior_prec)

Sigma[i, i] <- pow(sigma_par[i], 2)

sigma_par[i] ~ dunif(0, prior_ulim)

}

for(i1 in 1:(N_par - 1)) {

for(i2 in (i1 + 1):N_par) {

rho[i1, i2] ~ dunif(-1, 1)

Sigma[i1, i2] <- rho[i1, i2] * sigma_par[i1] * sigma_par[i2]

Sigma[i2, i1] <- rho[i1, i2] * sigma_par[i1] * sigma_par[i2]

}

}

beta4[1] ~ dnorm(0, prior_prec)

beta4[2] ~ dnorm(0, prior_prec)

log_lambda[1] ~ dnorm(0, prior_prec)

tau_lambda <- pow(sigma_lambda, -2)

sigma_lambda ~ dunif(0, prior_ulim)

}

**
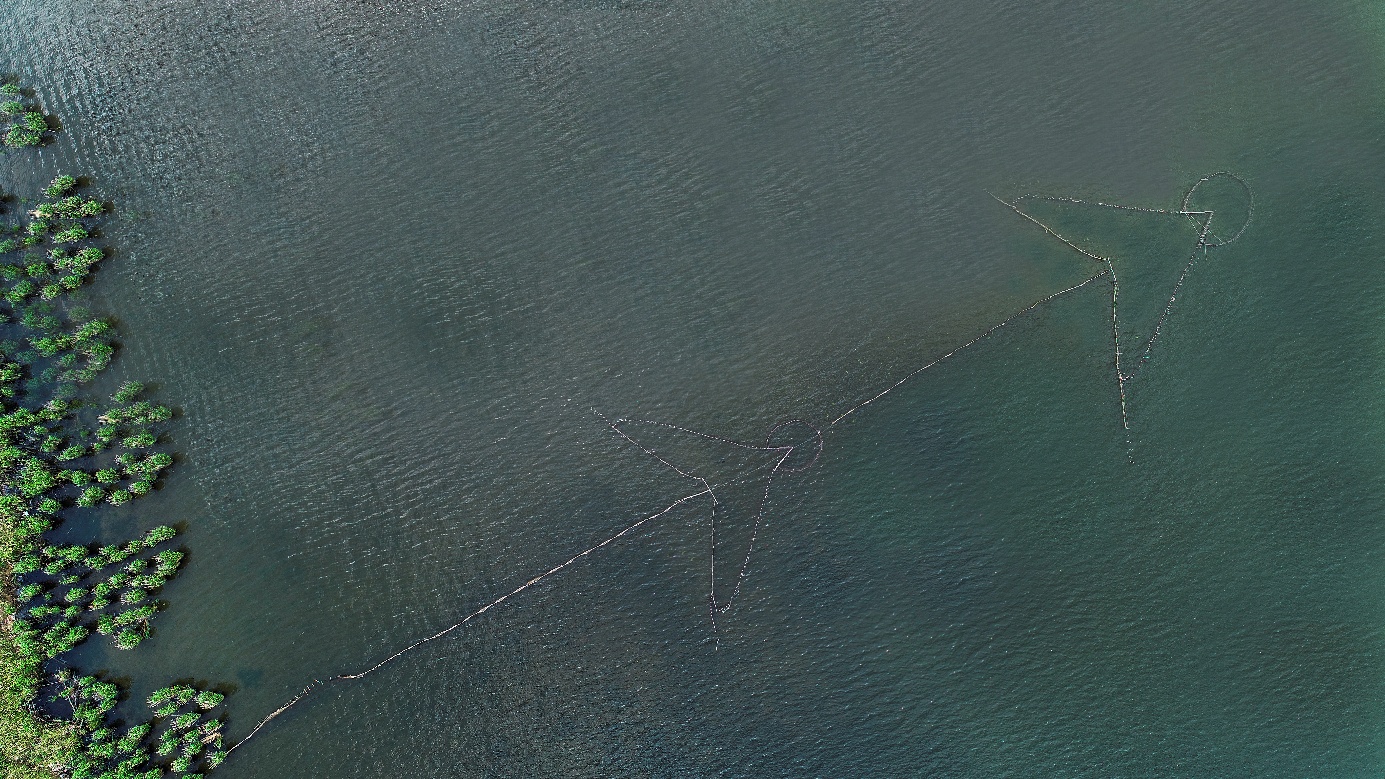
**

**
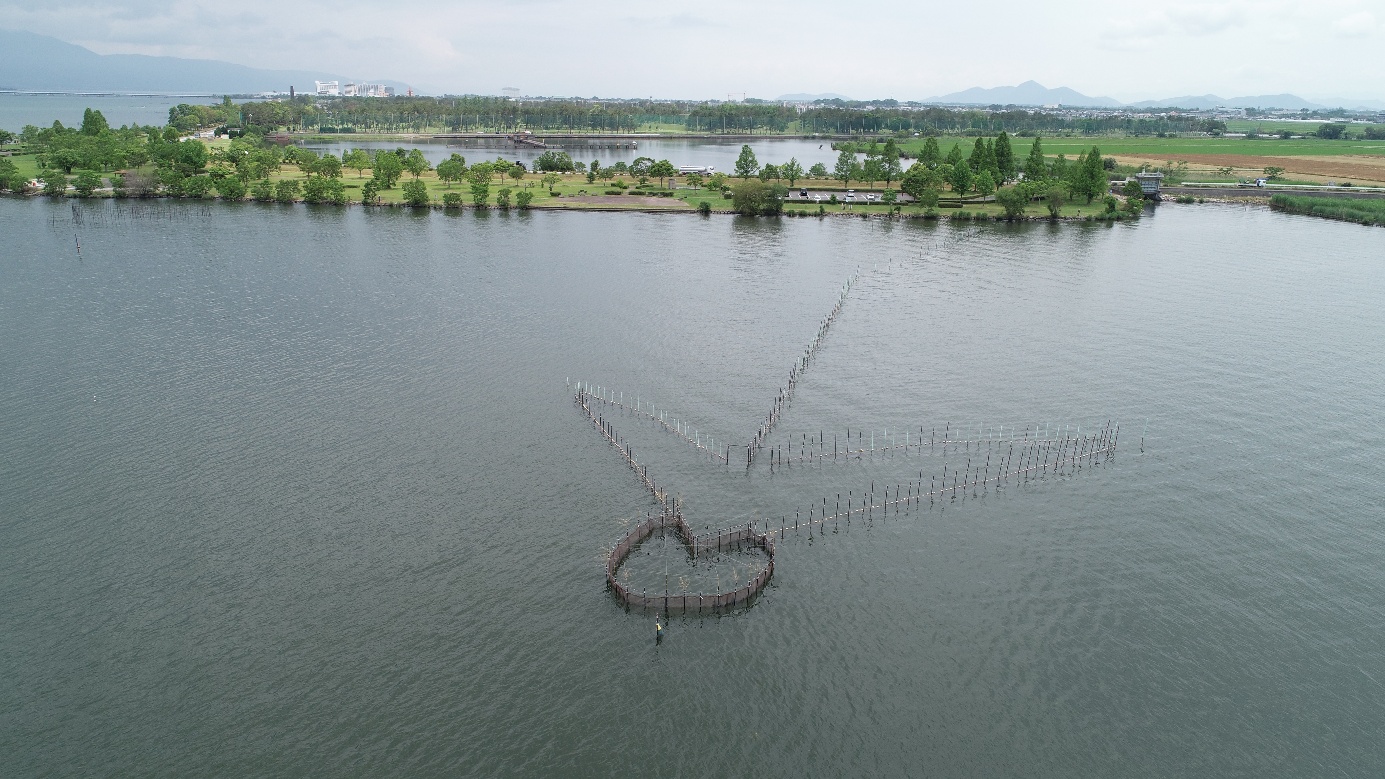
**

Fig. S1 Aerial pictures of *Eri*, a traditional, unique, large fixed-trap net that is used in Lake Biwa and has a history of approximately 1500 years. The *Eri*, which measure 50-200 m in length, are constructed so that they extend offshore from the lakeshore (For further details, see Kawanabe et al. 2012)

**Fig. S2** Relationships among candidate fishing effort indices. (a–c) There are positive correlations between the number of fishers and the number of Eri, the number of fisher households using Eri, and number of fisher households using gillnets (data from Shina Fisheries Cooperative Association) during 1966−2022. (d) There is a positive correlation between the number of fisher households and total annual fishing days in Kusatsu City, including the Shina, Yabase, and Yamada Fisheries Cooperative Association, during 1970−2001

**Fig. S3** Long-term changes of water level (1966−2022) in (a) May, (b) June, and (c) July, in Lake Biwa. Red vertical lines indicate the implementation of the new water-level regulation in 1992. B.S.L.; Lake Biwa Standard water level

**Fig. S4** Long-term trend of raw CPUEs (log-transformed) of total exotic fish species (largemouth bass and bluegill) in the south basin of Lake Biwa. Note that there are no catch records of total exotic fish species before 1982

**Fig. S5** (A) Trends of annual means of surface water temperatures in the south basin (1980–2022) and air temperatures at the Hikone Observatory (1966–2022). (B) Positive, significant relationship between annual mean surface water temperatures and annual mean air temperatures (1980–2022)
